# Supplementary material for: Electrochemical lateral flow assay with ELISA-level performance for detecting plant diseases in East Africa
Source: Proc Natl Acad Sci U S A. 2026 Jul 20;123(30):e2602947123. doi: 10.1073/pnas.2602947123 (PMC13416961; doi:10.1073/pnas.2602947123)
Supplement: Supplementary file 1 — Appendix 01 (PDF) [file pnas.2602947123.sapp.pdf]

Supplementary Information for

Electrochemical lateral flow assay with ELISA level performance for detecting plant diseases in East Africa

José M. R. Flauzino[1†], Abdulkadir Sanli[1†], Rudolph R. Shirima[2], Yuanjun Cai[1], Tinghao Hu[1], Leyang Li[1], Taliya Weinstein[1], Hong Seok Lee[1], Tayeb Tadlaoui[1], Selin Olenik[1,3], Abdülkadir Gümüşçü[1,4], Laura Gonzalez-Macia[1,5], George Mahuku[2], James Legg[2], Anthony E.G. Cass[6], Firat Güder[1,3*]

[1]Department of Bioengineering, Royal School of Mines, Imperial College London, SW7 2AZ, United Kingdom.

[2]International Institute of Tropical Agriculture, Dar-es-Salaam 14112, Tanzania

[3]Bezos Centre for Sustainable Protein, Imperial College London, London, SW7 2AZ, United Kingdom

[4]Department of Electrical and Electronics Engineering, Harran University, Şanlıurfa 63050, Türkiye

[5]Department of Life Sciences, University of Bath, Claverton Down, Bath, BA2 7AY, United Kingdom

[6]Department of Chemistry, Molecular Sciences Research Hub, Imperial College London, W12 0BZ United Kingdom

†These authors contributed equally to this work

*E-mail: guder@ic.ac.uk

AuNPs aggregation test. In each step of the AuNPs modification, a 200 μL aliquot was collected and placed in a 96-well plate. The aggregation of the nanoparticles was induced by applying 40 μL of a 1M solution of NaCl. The absorbance in the gold aggregation protocol was measured at 550 nm and 600 nm to assess the levels of aggregation in the wells. The higher the A550nm/A600nm rate, the more stable the colloid. As shown in Fig. S1 the colloid was more stable after the modification with the antibody, with no visible precipitation after the addition of salt.

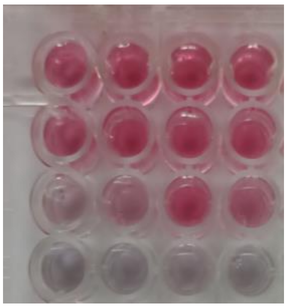

**Sample**	**A550nm/A600nm**			
AuNPs + Water	3.87	3.52	3.59	3.56
AuNPs/FcCOOH/AntiCBSV + NaCl 1M	2.62	3.25	3.53	3.39
AuNPs/FcCOOH + NaCl 1M	1.07	1.37	2.08	1.77
AuNPs + NaCl 1M	0.93	0.91	0.95	0.91

Figure S1: Photography of a 96 well plate with gold nanoparticles colloid modified with different molecules before (first row) and after (2nd to 4th rows) the addition of 1M of NaCl (n = 4).

ATR-FTIR characterization of Fc-AuNPs. ATR-FTIR spectroscopy was performed to provide evidence of AuNP functionalization with ferrocene carboxylic acid (FcCOOH). Three samples were analyzed: bare citrate-stabilized AuNPs, free FcCOOH powder, and Fc-AuNP conjugates (Fig. S2). For AuNP-containing samples, nanoparticle suspensions were concentrated by centrifugation prior to FTIR analysis to improve signal intensity. Briefly, AuNP suspensions were centrifuged at 5000 × g for 10 min, the supernatant was carefully removed, and the pellet was resuspended in deionized water to obtain concentrated nanoparticle suspensions. Fc-AuNP conjugates were prepared as described in the main Methods section and similarly concentrated before measurement. FTIR spectra were collected in ATR mode over the range of 4000–400 cm^{-1}, using a resolution of 4 cm^{-1} and 64 scans per spectrum. A fresh background spectrum was collected from the clean ATR crystal before each measurement. Free FcCOOH powder was placed directly onto the ATR crystal

and measured under pressure contact. For AuNP-containing samples, 5 μL of concentrated nanoparticle suspension was drop-cast onto the ATR crystal and allowed to dry at room temperature. This drop-casting step was repeated five times in the same position to build a sufficiently dense dried film for analysis. The ATR crystal was cleaned thoroughly with ethanol between samples, and background spectra were recorded to confirm removal of residual material. The resulting spectra are shown in Figure S2. Bare AuNPs showed weak citrate-associated features, whereas free FcCOOH displayed characteristic ferrocene-related bands. The Fc-AuNP spectrum retained ferrocene-associated bands, including features assigned to Fe–Cp and cyclopentadienyl ring vibrations. In addition, a band at approximately 1580 cm^{-1} was observed in the Fc-AuNP spectrum but was absent from both bare AuNPs and free FcCOOH. This band was assigned to amide II vibration, consistent with amide bond formation between FcCOOH and the cysteamine-modified AuNP surface via EDC/NHS coupling. The shifts in ferrocene-associated bands relative to free FcCOOH further support a change in the chemical environment of FcCOOH after conjugation. Together, the ATR-FTIR data support successful functionalization of AuNPs with FcCOOH.

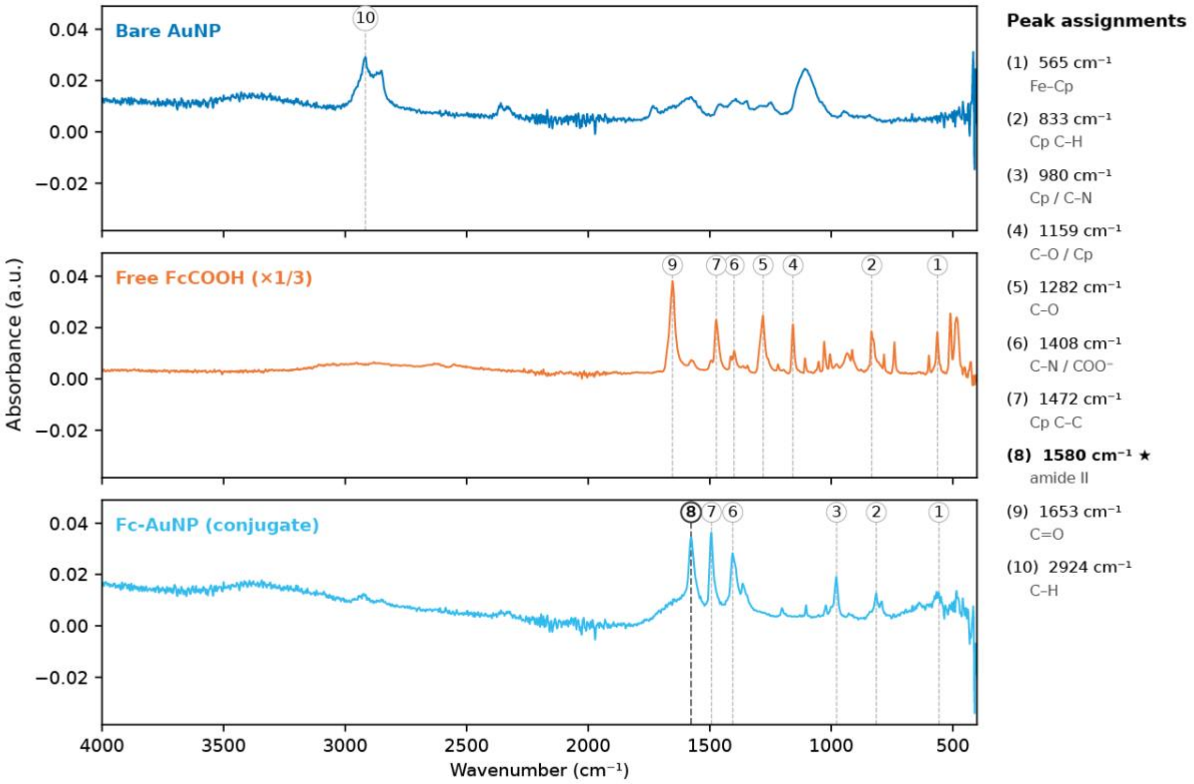

Figure S2 - FTIR spectra of bare AuNps, free FcCOOH and Fc-AuNPs

Hardware Design. We calculated the theoretical inductance value of a one-layer circular inductor using the Texas Instruments custom coil designer tool[1]. With an outer diameter of inductor (D_{out}) of 15 mm, a trace width (w) of 0.25 mm, a distance between traces (s) of 0.15 mm, 10 turns per layer (n) and a copper thickness of 1 oz-cu (~0.0347 mm). The resulting theoretical inductance value is 1.322 µH. This single-layer circular coil design achieves a quality factor greater than 50. As the resonance frequency for the near-field communication (NFC) tag is 13.56 MHz, we can easily calculate the external capacitor using the following formula:

$$f_{res} = \frac{1}{2\pi\sqrt{LC_{tun}}}$$

where f_{res} is the resonance frequency, L is the inductance of the antenna, and C_{tun} is the tuning capacitor, which is calculated as 104.205 pF. Since the internal capacitor of the SIC4341 is 50

pF, the required external capacitor would be 54.205 pF ($C_{ext} = C_{tun} - C_{int}$). We selected a 56 pF external capacitor, as it was the closest available option on the market.

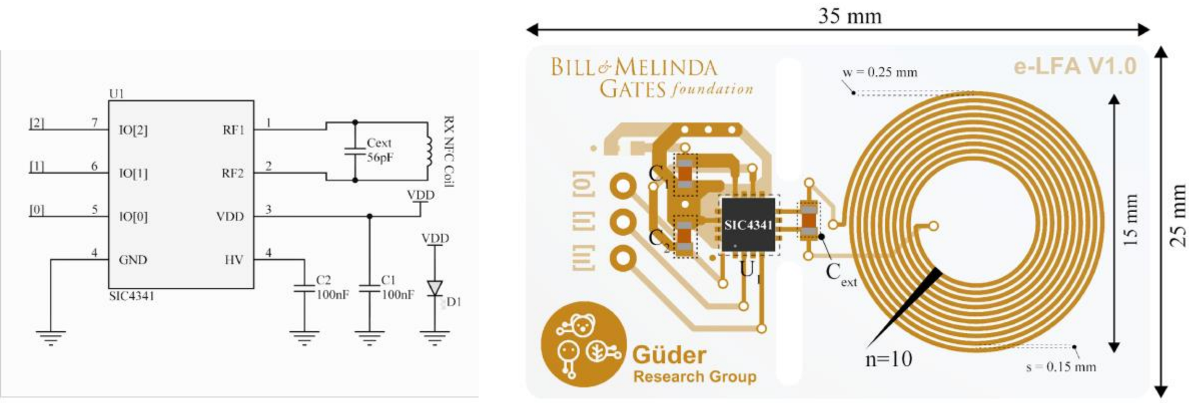

Figure S3: Schematic (left) and 3D PCB design (right) illustrating the receiver coil configuration. The schematic presents the functional components and connectivity of the receiver coil, while the 3D design highlights key design properties including dimensions, coil orientation, and layout on the PCB.

The SIC4341 NFC microchip incorporates an internal potentiostat designed to measure currents from chemical sensors, with a maximum input range of ± 20 µA. This chip can generate a biasing voltage between -800 mV and 800 mV for the working and reference electrodes, using dual digital-to-analog converters (DACs) with a 1.28 V full-scale voltage and 5 mV resolution. Our custom-made mobile application controls the biasing voltage of the sensor by sending commands to adjust the DAC output. The sensor current is processed by a 10-bit analog-to-digital converter and a digital signal processor and then stored in internal memory for transmission back to the smartphone.

To enhance user experience and provide visual feedback, we integrated an LED indicator into the PCB that illuminates when a stable NFC connection is established between the smartphone and the ELLA device. This feature improves usability and aids in troubleshooting. We printed the ELLA test cassettes using translucent Polylactic acid (PLA) filament, allowing users to observe the LED and receive real-time feedback on NFC connectivity while maintaining our commitment to sustainability. To ensure measurement consistency, we also designed a specialized 3D-printed phone case (demonstrated with a

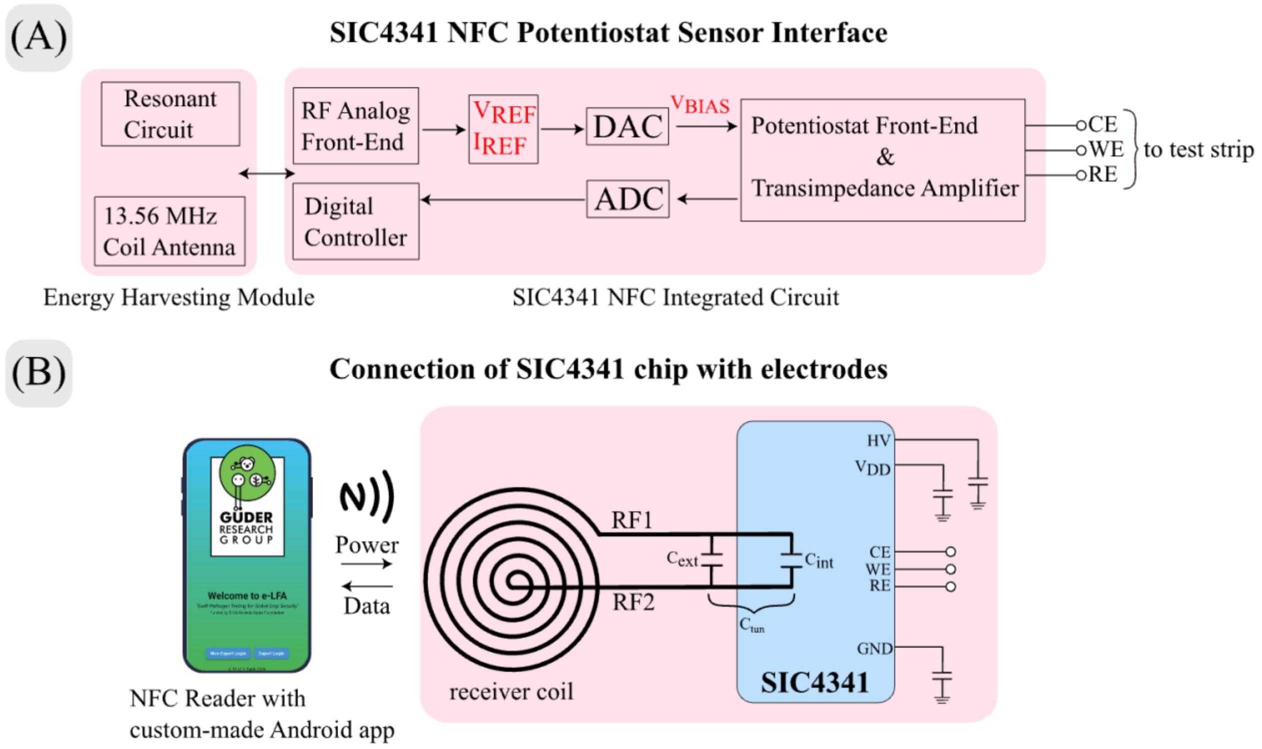

Figure S4: (A) The SIC4341 NFC potentiostat sensor interface, highlighting the data flow and interface setup. This figure illustrates how the SIC4341 chip, equipped with DACs, ADC, and RF AFE, communicates with connected electrodes, showing critical signal pathways and connection points for efficient data transmission. (B) Demonstrates the NFC wireless communication between a smartphone and the SIC4341 chip connected to the electrodes, facilitating seamless data transfer and power supply through NFC technology.

Huawei P30 Lite) that optimizes ELLA cassette placement and maintains uniform power distribution. These enhancements collectively improve the functionality and user experience of the ELLA device, providing clear visual cues for successful communication and facilitating proper device operation.

Software Development. We developed a user-friendly custom-made app interface, where we have meticulously designed two login pages tailored for "non-expert" and "expert users" (see Fig. S5). The non-expert page prioritizes simplicity, featuring a detailed instructional guide to assist users in conducting precise measurements. Upon insertion of the test cassette and

initiation of the measurement process via the START button, users are prompted to input basic details such as username, file name, and type of virus (i.e., CBSV, BBTV, etc.), after which the app autonomously progresses through calibration, conversion, and result retrieval. The "expert page" on the other hand enables the user more flexibility in parameter customization, allowing selection between voltammetric (i.e. square wave voltammetry, cyclic voltammetry) or amperometric (chronoamperometry) measurements. Notably, ELLA operates seamlessly both online and offline, leveraging MongoDB Atlas cloud Database for online functionalities. This integration enables users to monitor, and share collected data, alongside a Database Map illustrating virus spread over time. Additionally, all measurements are catalogued in a calendar format for easy reference.

The cloud-based storage of measurement data enhances the functionality of the mobile app by allowing users to share results through various communication channels. Users can easily distribute their findings via email, SMS, or other messaging platforms, facilitating rapid information dissemination among researchers, healthcare professionals, or relevant stakeholders. This feature promotes collaboration and enables quick response to potential outbreaks or emerging trends in virus detection. The combination of user-friendly interfaces, flexible measurement options, and robust data-sharing capabilities makes ELLA a comprehensive tool for both field diagnostics and advanced laboratory analysis.

We also designed a custom 3D-printed PLA phone case incorporating a cassette housing aligned with the smartphone NFC coil (Huawei P30 Pro, located on the rear upper-middle region). The housing ensures reproducible lateral positioning without adding extra thickness. The total distance between the NFC coil and the cassette antenna is approximately 3.5–4.2 mm. This controlled geometry improves coupling stability and measurement reproducibility (Fig S5c).

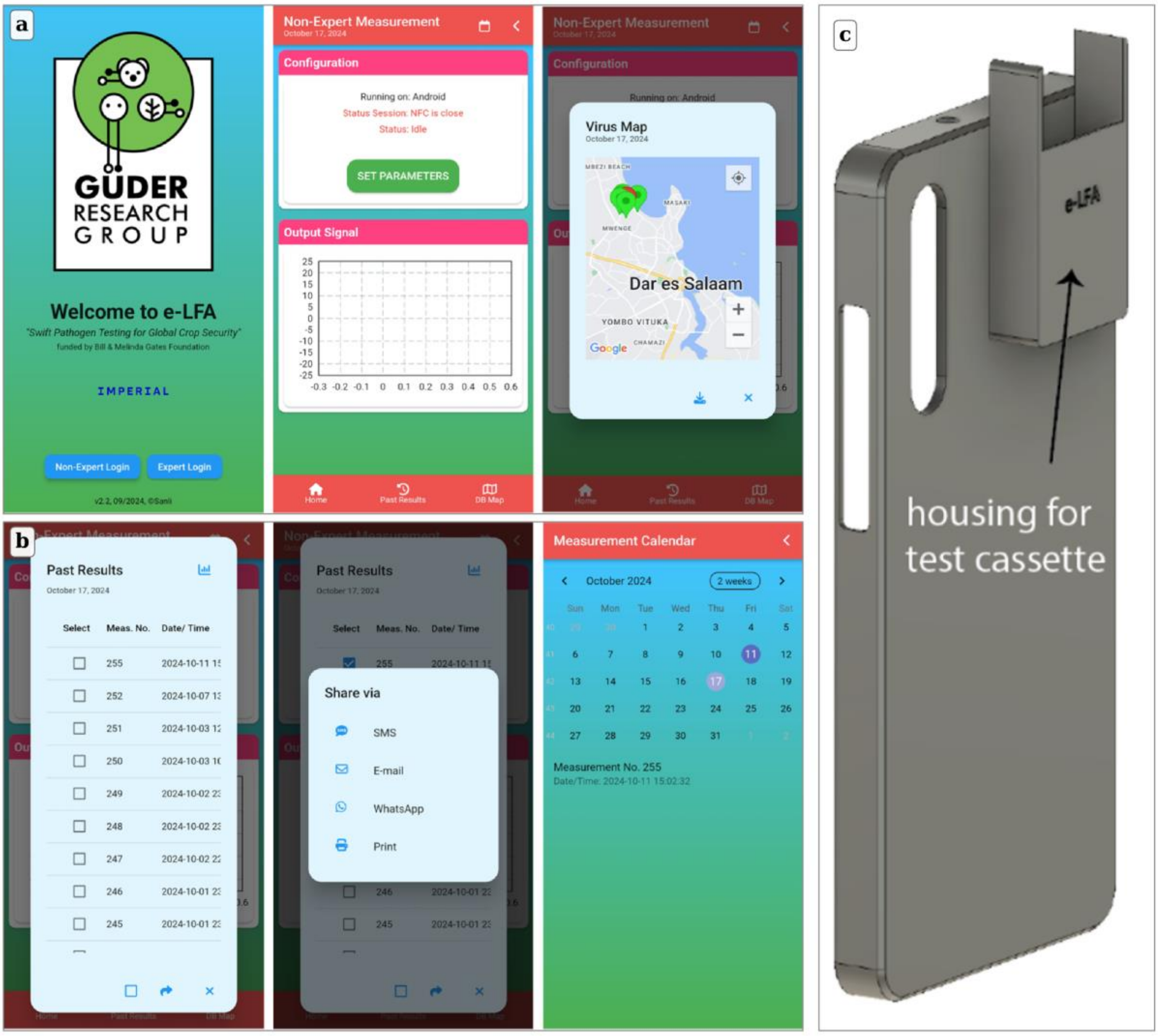

Figure S5: Screenshots of the custom-designed mobile app interface, developed with SIC4341 libraries in Flutter using the Dart programming language on Android Studio. (A) The configuration page allows users (expert and non-expert) to input settings and view a real-time virus map, which is automatically updated from a MongoDB database. This page provides essential controls for configuring device parameters, ensuring ease of use across different skill levels. (B) The measurement page presents a comprehensive list of all recorded measurements retrieved from both local and cloud-based databases, with options for data sharing and printing. A measurement calendar enables users to select specific dates to access and review individual measurements. This database system serves as a centralized repository for data sharing, encompassing experimental results, location-based information, and other relevant metrics, allowing seamless collaboration and broad accessibility across platforms. (C) Phone case designed for the measurements to ensure alignment of the device with the NFC antenna of the phone.

Electrodes characterization. Scanning electron microscopy (SEM) of the gold-plated acupuncture needles revealed a smooth and uniform surface morphology along the shaft, with occasional microscopic imperfections and pits likely originating from the gold coating or

underlying stainless-steel substrate (Fig. S6). These minor surface defects are typical of commercially fabricated acupuncture needles and did not appear to disrupt the overall coating integrity. The absence of significant roughness or porosity suggests that the electrode surface is well-suited for reproducible electron transfer. No delamination or major inconsistencies were observed across multiple needles, supporting the use of these low-cost materials as reliable and consistent electrochemical interfaces.

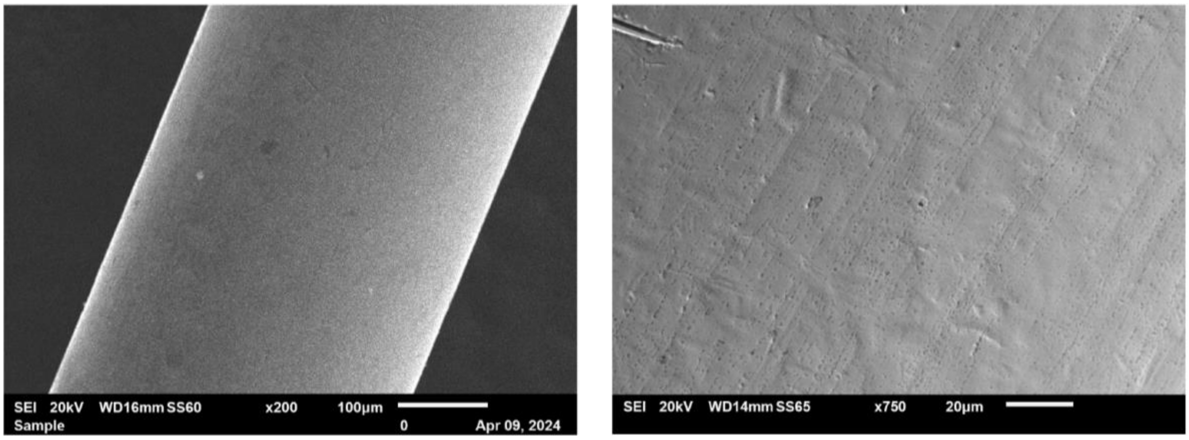

Figure S6: Scanning electron microscopies of the needles used as electrodes.

Cyclic voltammetry (CV) was carried out to characterize the electrochemical behavior of screen-printed gold electrodes (SPGEs) and the acupuncture-needle electrodes (Fig. S7). A 2 mM solution of hexaammineruthenium(II/III) chloride (RuHex) in phosphate-buffered saline (PBS) was used as the redox probe. For the needle configuration, a silver-plated acupuncture needle served as the reference electrode, while gold-plated acupuncture needles were used as the working and counter electrodes; the needles were directly connected to a PalmSens 4 potentiostat. All electrodes (working, reference, and counter) were immersed 1 cm into the electrolyte solution. CVs were recorded at scan rates of 1000, 500, 250, 100, 50, 25, 10, and 5 mV s^{-1} between -0.5 V and 0.1 V. We selected RuHex because, when using ferri/ferrocyanide under the same conditions, we consistently observed a triangular (clipped) oxidation peak and, especially at low scan rates, and a pronounced potential drift; RuHex provided stable, well-

defined responses across the entire scan-rate range.

The needle-based electrodes exhibited a strong linear relationship between peak current and the square root of the scan rate, with $R^2 = 0.998$ for oxidation and $R^2 = 0.995$ for reduction, indicating highly reversible and diffusion-limited electron transfer. For comparison, commercial screen-printed gold electrodes (SPGEs 220AT, Metrohm-Drop Sens) tested under the same conditions showed similarly high correlation coefficients ($R^2 = 0.999$ for oxidation and $R^2 = 0.997$ for reduction) and nearly identical redox peak positions. These observations confirmed that both systems display near ideal electrochemical behavior with the $[Ru(NH_3)_6]^{3+/2+}$ probe. Using a diffusion coefficient of 7.1×10^{-6} $cm^2{\cdot}s^{-1}$ for $[Ru(NH_3)_6]^{3+}$, [2]we estimated the electroactive surface area for the Au needle electrodes from the slope of the Randles–Ševčík plots. The needle electrodes yielded a calculated electroactive area of 7.1 mm^2, while the SPGE electrodes had a lower electroactive area of 4.5 mm^2 although both types of electrodes had the same geometric area (12.5mm^2) in contact with the electrolyte solution. This discrepancy suggests that a smaller portion of the SPGE surface is electrochemically active, potentially due to the presence of non-conductive components in the ink, whereas metallic continuity of the needles likely provided a more accessible, electroactive surface for the redox reactions. Overall, the needle electrodes demonstrated superior electrochemical performance, making them the preferred choice for integration into the ELLA cassette.

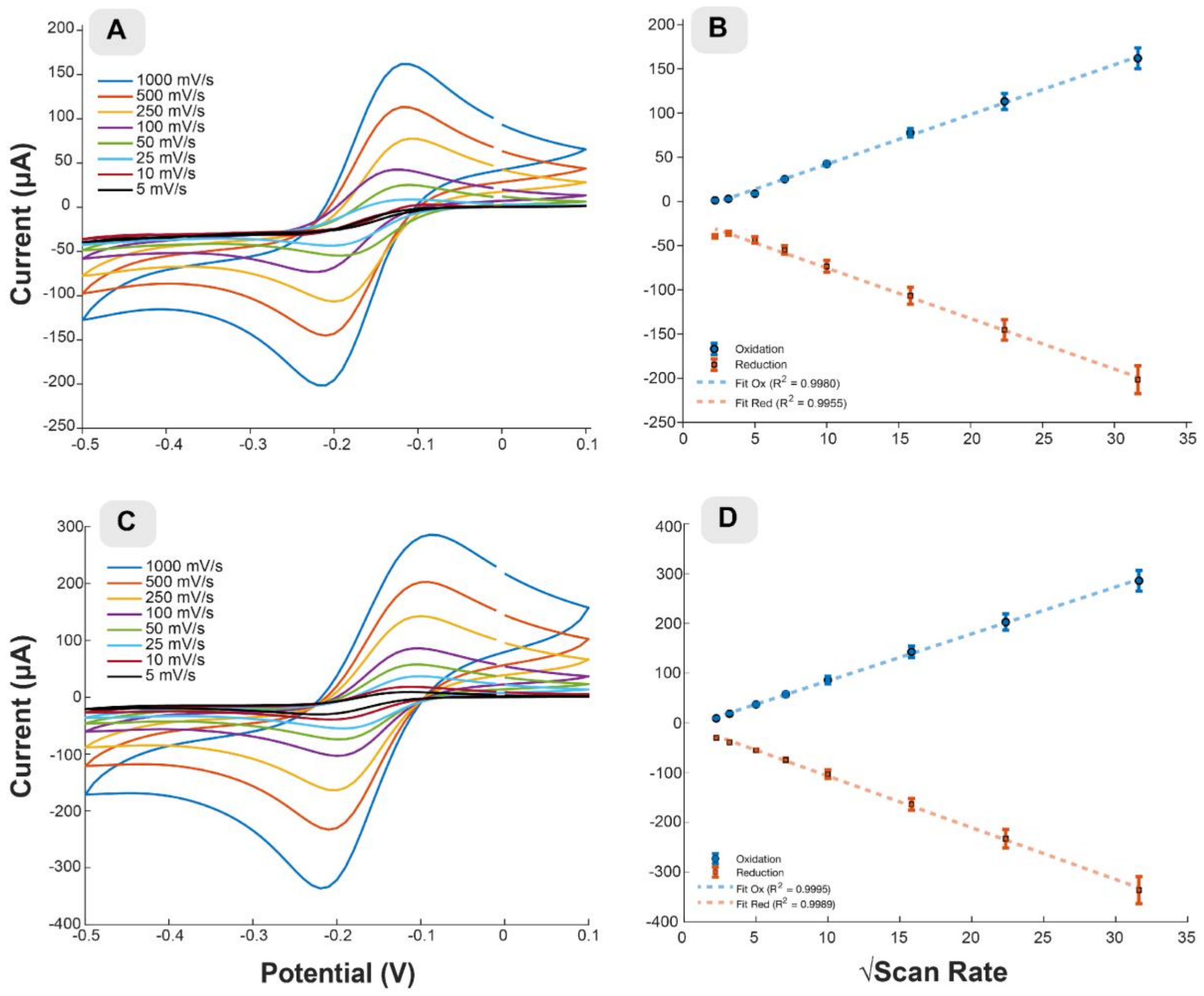

Figure S7: (A) Cyclic voltammograms of gold acupuncture needle electrodes recorded at scan rates of 5, 10, 25, 50, 100, 250, 500, and 1000 mV·s^{-1} in 2 mM hexaammineruthenium(III)/(II) chloride dissolved in PBS (pH 7.4). The electrochemical cell consisted of gold-plated acupuncture needles as the working and counter electrodes, and a silver-plated acupuncture needle as the pseudo-reference electrode. All electrodes were immersed 1 cm into the electrolyte and connected to a benchtop potentiostat. **(B)** Corresponding peak anodic and cathodic currents plotted as a function of the square root of the scan rate, demonstrating linearity consistent with diffusion-controlled electrochemical behavior. **(C–D)** Identical measurements and analysis performed using commercial screen-printed gold electrodes (SPGEs), showing comparable voltammetric profiles and peak current dependence on scan rate. All potentials are versus Ag^0 silver ink.

Open-circuit potential stability of the silver needle quasi-reference electrode Open-circuit potential measurements were performed to evaluate the short-term stability of the silver-plated stainless-steel acupuncture needle used as a quasi-reference electrode in the ELLA platform. Measurements were carried out in a fresh CBSD extraction buffer for each replicate. A commercial Ag/AgCl electrode was used as the reference electrode, and the silver-plated

acupuncture needle was used as the working electrode. The silver needle was fixed vertically using a jig to ensure a constant immersion depth of 3 mm. The potential difference between the silver needle and the commercial Ag/AgCl reference electrode was recorded every second using a PalmSens 4 potentiostat. Measurements were performed for 25 min, corresponding to the maximum assay time used in the ELLA workflow, and repeated three times. The OCP measurements are shown in Figure S8. The potential mean shift was -38.6 mV over 25 min. Although this confirms that the silver-plated needle behaves as a quasi-reference rather than a true Ag/AgCl reference electrode, the drift was considered acceptable for the short-duration, single-use ELLA assay. This is because ELLA relies primarily on peak-current thresholds under standardized assay conditions rather than precise absolute potential determination.

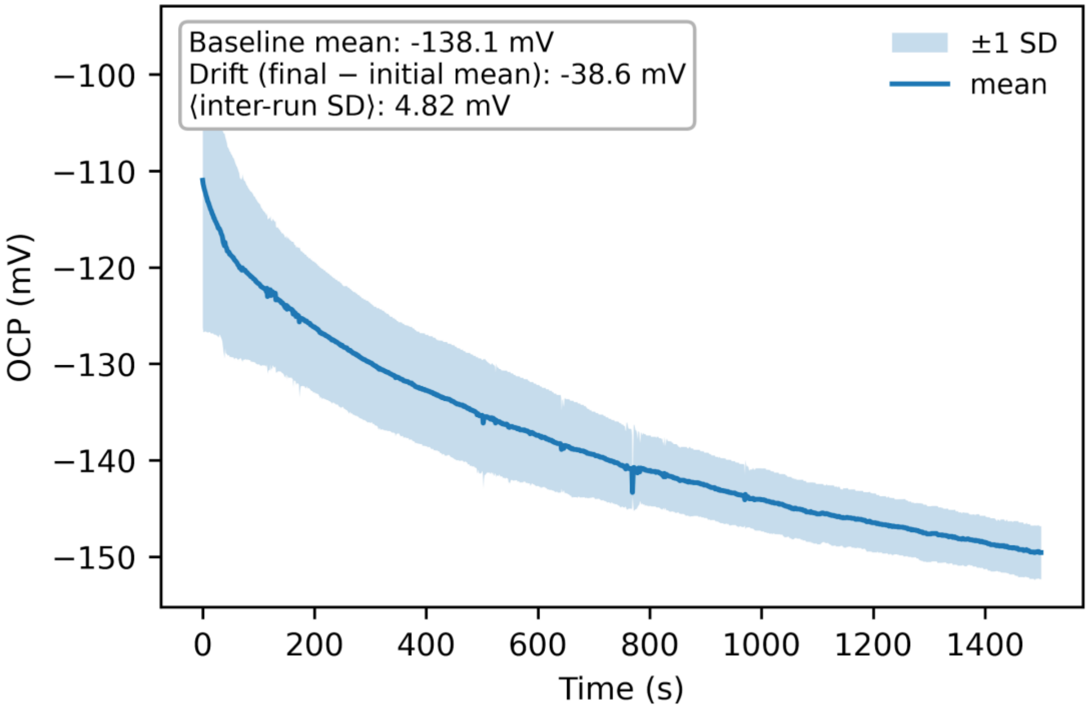

Figure S8 - Open circuit measurement of the silver needle against a Ag/AgCl reference electrode (n = 3).

Platform comparison. To evaluate the analytical performance of the custom-built PCB potentiostat relative to the commercial benchtop system (Autolab PGSTAT204, Metrohm), square-wave voltammetry (SWV) was performed in different concentrations of potassium

ferri/ferrocyanide ($Fe(CN)_6^{3-/4-}$), dissolved in phosphate-buffered saline. For both instruments, identical three-electrode configurations were employed, ensuring the same geometric electrode area and electrolyte volume. The SWV parameters were: range from -0.8 to +0.8, amplitude 50mV, frequency 2.5 Hz. The peak current was extracted and averaged for each concentration. Calibration curves were generated (Fig. S9) by plotting the mean peak current against analyte concentration, with error bars representing the standard deviation ($n = 3$). The resulting datasets were fitted with logarithmic regression models, yielding coefficients of determination (R^2) of 0.975 and 0.976 for the PCB and Autolab systems, respectively. The fitted equations were ip = 5.1403ln(x) + 10.1730 for the PCB and ip =5.1605ln(x) +1 1.8925, where ip is the SWV peak current (μA) and x the $Fe(CN)_6^{3-/4-}$ concentration (mM). Both instruments exhibited a similar concentration-dependent response, characterized by a steep current increase at low concentrations followed by mild saturation above 2.5 mM, consistent with diffusion-limited behavior of the redox couple. The PCB potentiostat consistently produced currents approximately 17% lower in magnitude than the Autolab, attributable to differences in front-end amplification and current scaling; however, the sensitivity trend and non-linear shape were effectively identical. These results confirm that the PCB potentiostat reproduces the electrochemical response of the commercial instrument with fidelity, validating its use as an embedded readout unit for the ELLA platform.

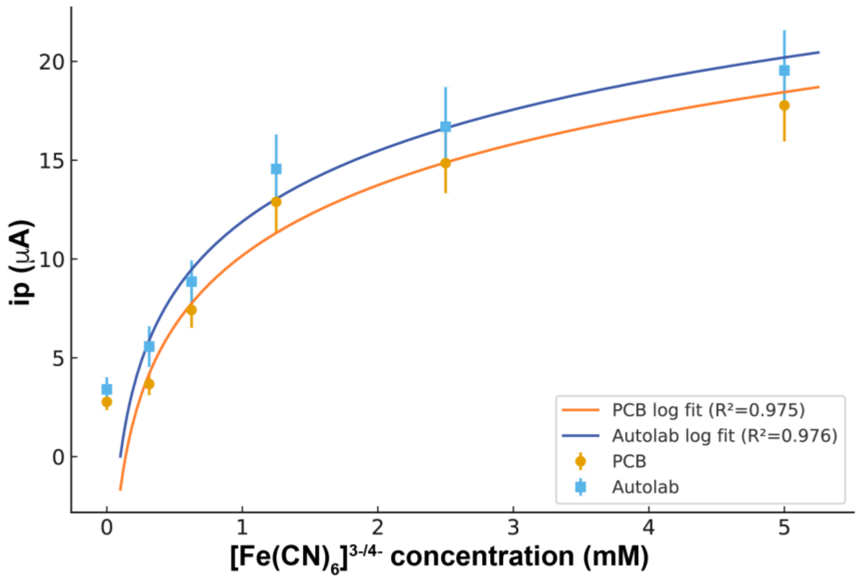

Figure S9: Comparison of calibration curves obtained using the custom PCB potentiostat and a commercial Autolab system. Square-wave voltammetry (SWV) peak currents recorded for increasing concentrations of ferri/ferrocyanide in PBS (0–5 mM) were fitted with a logarithmic function (potential range from -0.8 to +0.8, amplitude 50mV, frequency 2.5 Hz, n = 3)

Assembly process of test strips for ELLA. The fabrication of the test strips involves preparing the nitrocellulose membrane and assembling key components, including the absorbent pad, nitrocellulose membrane, and the sample + AuNPs conjugate pad. Fig. S10 showcases both pre-test and post-test views, highlighting the appearance of a positive result.

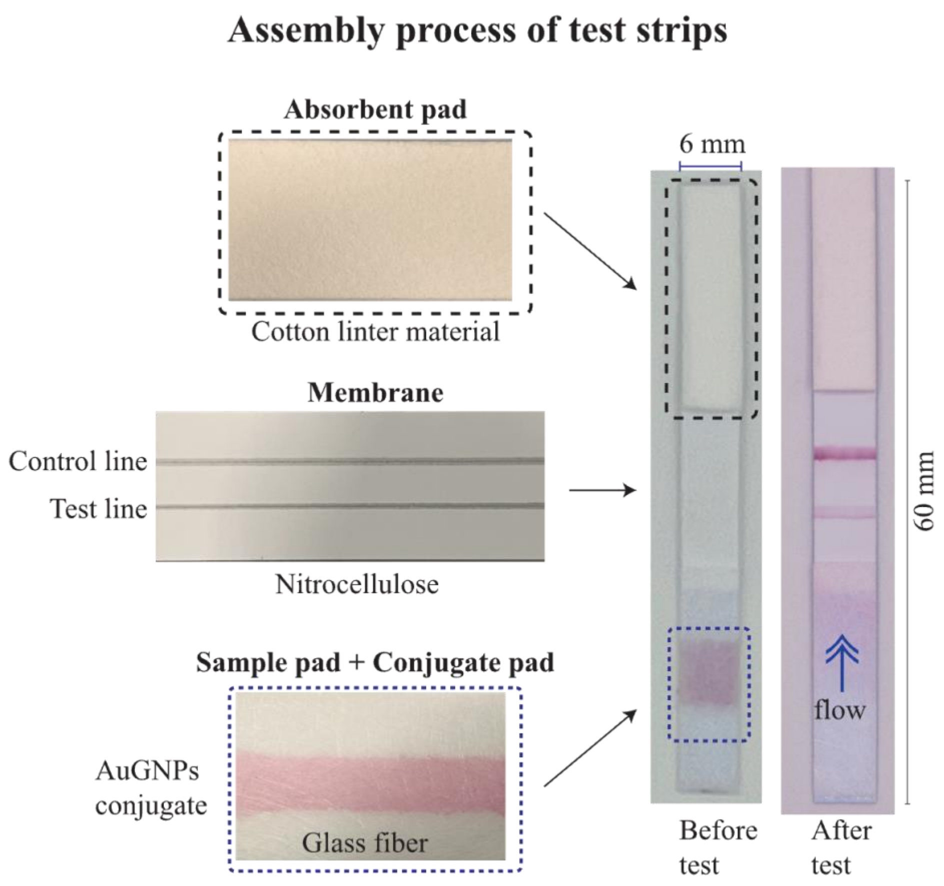

Figure S10: Assembly process of the test strips, including the preparation of the nitrocellulose membrane and the assembly of the test strip components: the absorbent pad, nitrocellulose membrane, and sample + AuNPs conjugate pad. Both pre-test and post-test views are shown, demonstrating the appearance of a positive result.

Design of a 3D printed custom-made hole puncher device. We designed a custom 3D-printed puncher that simultaneously aligns the nitrocellulose test strip with the PCB, which already carries the soldered needle electrodes (Fig. S11). In a single operation, the puncher perforates the strip and positions the electrodes through the membrane just after the test line, maintaining the structural integrity of the paper and ensuring precise, reproducible placement for subsequent measurements. The tip of the needle can be modified to ensure residue-free holes.

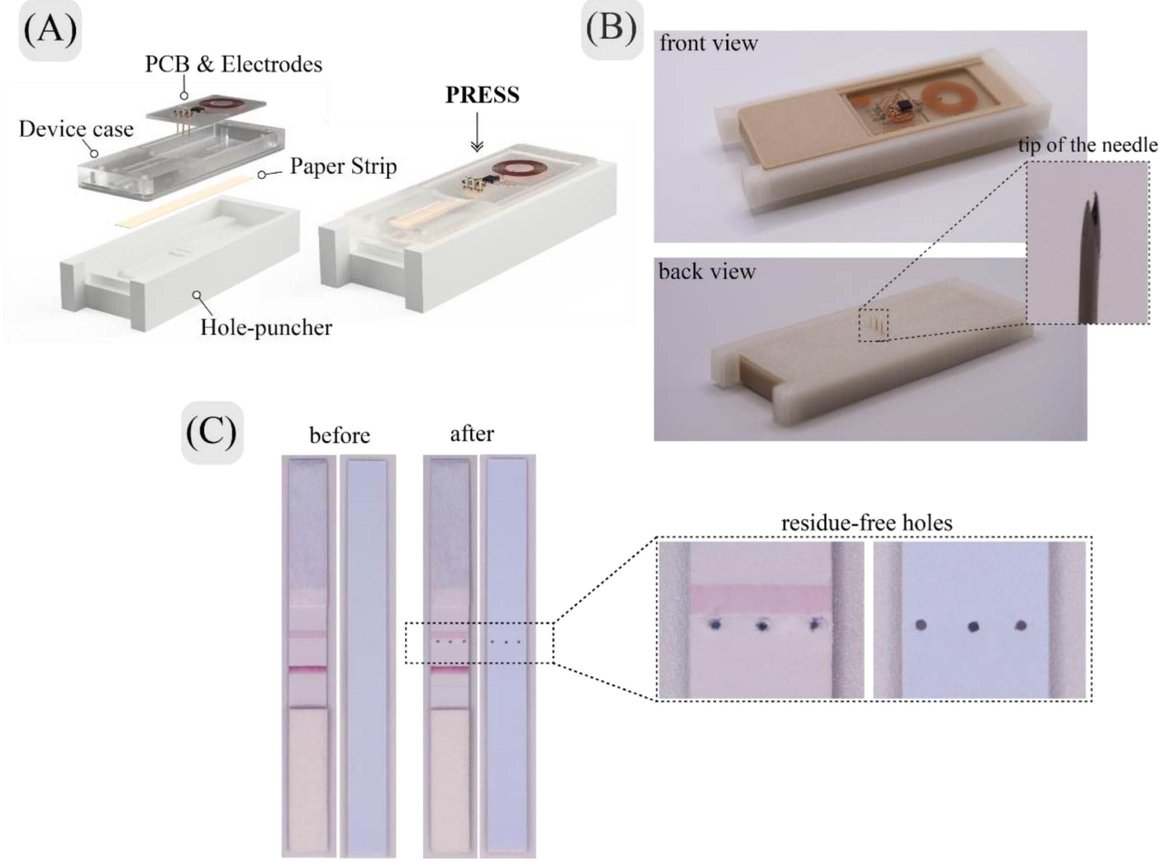

Figure S11: (A) 3D sketch and (B) photograph of the custom-designed hole puncher device, illustrating the integration of three curved-tip needles for precise perforation of nitrocellulose paper. (C) Front and back views of the punched sample demonstrate the device's capability to create consistent, residue-free holes, enhancing measurement reliability.

Fc-AuNP calibration curve. To assess the relationship between electrochemical signal and Fc-AuNP amount, a calibration experiment was performed using Fc-AuNP suspensions prepared at different optical densities. Fc-AuNP solutions were diluted to OD 1, 2, 3, 4, and 5 and measured under the same square-wave voltammetry conditions used for the ELLA platform (Fig S12 a-c). Measurements were performed using a 3D-printed LFA holder and needle aligner to ensure reproducible electrode placement. The needle electrodes were positioned 2 mm apart and inserted vertically into the nitrocellulose strip using a fixed mechanical stop to ensure consistent puncture depth (Fig S12). Each test used a fresh 6 mm nitrocellulose strip adhered to a backing card. Sample and wicking pads were not included, as the objective was to evaluate the electrochemical response of the Fc-AuNPs under static

conditions without capillary flow. For each measurement, 40 µL of Fc-AuNP suspension was applied to the nitrocellulose membrane. Square-wave voltammetry was performed using a Metrohm Autolab PGSTAT204 potentiostat with the following parameters: potential range from −0.3 V to +0.6 V, amplitude of 25 mV, and frequency of 5 Hz. A new nitrocellulose strip and a new set of needle electrodes were used for each measurement. The resulting calibration curve is shown in Figure S12d-e. The SWV peak current increased with Fc-AuNP optical density, confirming that the electrochemical response scales with the amount of electroactive nanoparticle reporter present on the strip.

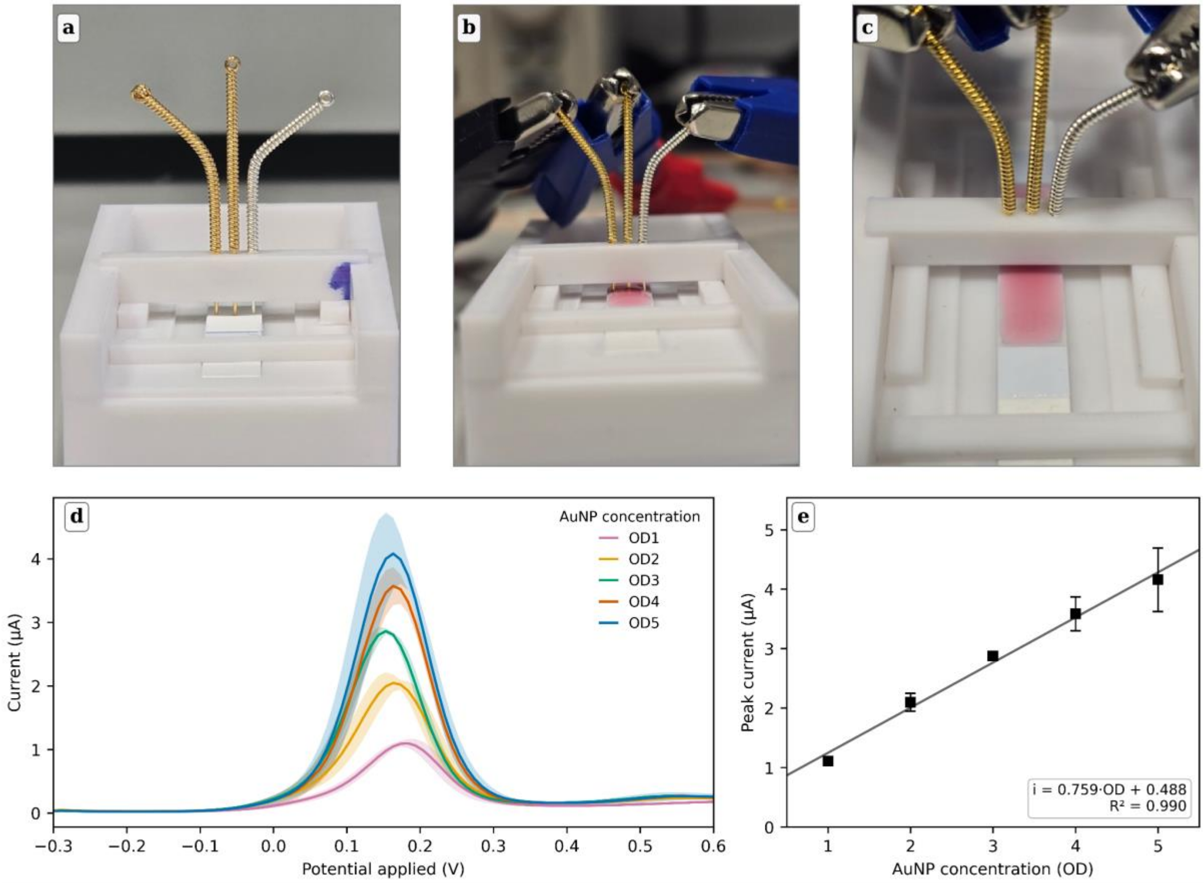

Figure S12 - Fc-AuNP calibration curve: (A-C) photos of the setup used for making the calibration curve onto the strips. The 3 electrodes (gold-plated needles for working and counter and silver needle for pseudo-reference electrode) are connected to the potentiostat. A 3D-printed case aligns the electrodes onto the strip. (D) Square wave voltammograms of dilutions of Fc-AuNPs, all potentials are versus Ag pseudo-reference electrode. (E) Calibration curve with the resulting linear regression (n = 3).

Stability test. To assess the shelf-life of the ELLA cassettes, the devices were stored at room temperature (25 °C ± 1), protected from light and sealed in metallized/Mylar accompanied by silica gel pouches to maintain low humidity levels. The stability study was conducted over a 5-month period, during which the ELLA cassettes were tested monthly using a positive control sample to monitor performance consistency. The results revealed a gradual decline in current, with current readings exhibiting a linear decrease, particularly noticeable from the second month of storage onwards. This decline suggests potential degradation of the active components or changes in the electrochemical properties over time. These findings highlight the importance of further optimization in the storage protocol or component stability to extend the effective shelf-life of the devices.

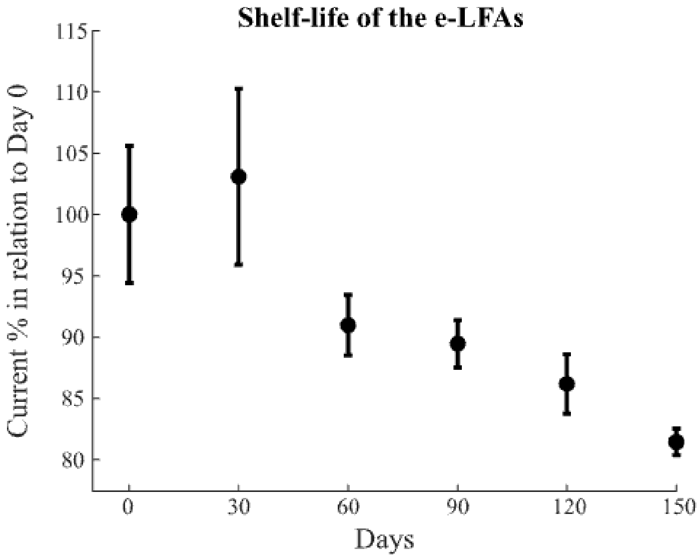

Figure S13: Shelf-life evaluation of ELLAs over a 5-month storage period. Devices were stored at 25°C, protected from light, and paired with silica pouches to maintain a dry environment. Performance was assessed monthly using a positive control sample to monitor functionality and stability over time.

AI Classification of SWV. SWV data were collected from 125 independent measurements. Each voltammogram was processed to extract both statistical and signal-shape features for machine learning classification. The following features were computed: maximum and

minimum current, mean and standard deviation of current, area under the current–potential curve, maximum and minimum of first derivative (slope), skewness of the current distribution, peak potential, peak prominence, fast Fourier transform (FFT) descriptors, and polynomial fitting coefficients. Feature correlation analysis was performed to identify independent contributors. From the initial set, 13 features were found to provide non-redundant information. The most relevant among these included maximum/minimum current, skewness of the current shape, peak potential, peak width, FFT-derived descriptors, and polynomial coefficients. Feature importance was estimated by training ensemble models and ranking their contribution to classification accuracy (Fig. S10). Based on this analysis, the eight most informative features were selected for model development. Five supervised learning algorithms were evaluated: logistic regression, random forest, extreme gradient boosting (XGBoost), support vector machine (SVM with RBF kernel), and a multilayer perceptron (MLP, feed-forward neural network). For each model, hyperparameters were optimized through grid/random search where applicable. Model performance was assessed using repeated stratified k-fold cross-validation (k=5, 10 repeats), with 80% of data used for training and 20% held out for testing. All models achieved broadly comparable performance, with test set accuracies around 83% and ROC AUC values between 0.83–0.86. This performance was lower than that obtained using a simple thresholding approach on peak current alone, as reported elsewhere in this study. Nonetheless, the machine learning analysis demonstrates that SWV data contain rich, multidimensional information that can be leveraged for classification, and may yield further improvements with larger datasets or advanced feature engineering.

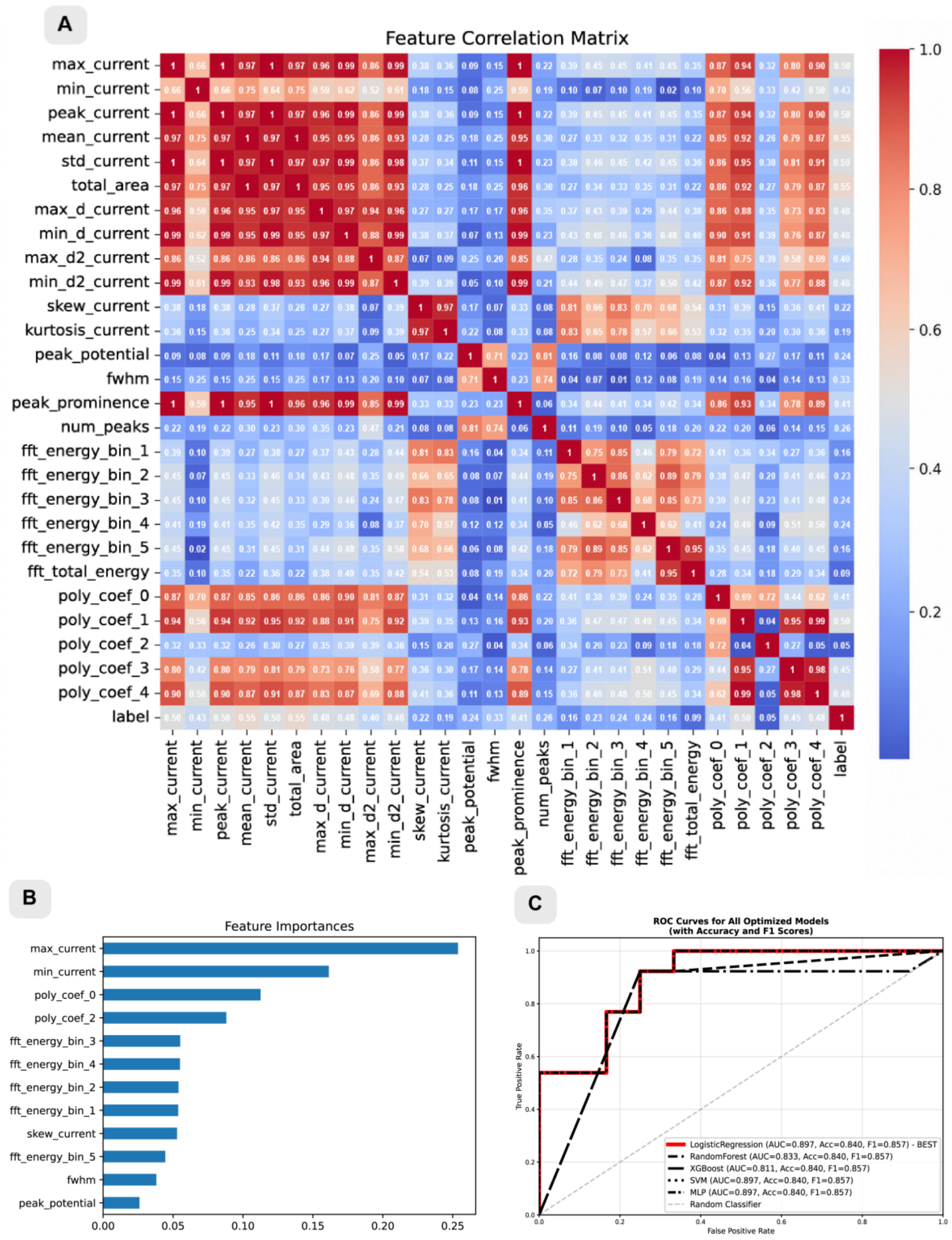

Figure S14: Feature evaluation and performance of machine learning models for CBSV detection. (**A**) Feature correlation matrix of the processed dataset (27 manually-derived features from SWV data), constructed from the absolute Pearson correlation coefficients among all extracted features. The heatmap highlights redundancy and potential multicollinearity, which were used to guide feature reduction by removing highly correlated variables (> 0.9). (**B**) Feature importance analysis using a Random Forest classifier trained on the reduced dataset. Feature importance values were calculated from the mean decrease in Gini impurity, providing a relative ranking of each

feature's contribution to model decision-making. The two most-important features were selected for model training as increasing the number had no effect or deteriorated model performance. (**C**) Receiver operating characteristic (ROC) curves generated for all optimized models following stratified train–test splitting (80/20). Hyperparameter optimization was performed on five models (logistic regression, random forest, XGBoost, support vector machine, and multilayer perceptron) using grid search with stratified 5-fold cross-validation on the training set to identify the parameter set yielding the best mean performance. The final models were then retrained with the selected hyperparameters and evaluated on the test set. ROC curves are shown alongside accuracy and F1 scores for each model.

ELISA and RT-qPCR tests. The ELISA kit for detecting CBSV was obtained from the Leibniz Institute DSMZ (Germany) and was conducted according to the recommended guidelines. Briefly, it involves a triple antibody sandwich format. The process begins with coating 96-well plates with purified IgG against CBSV (AS-1153) diluted in coating buffer, followed by incubation and washing. A blocking step with 2% skim milk in PBS-Tween is performed to prevent nonspecific reactions. Leaf samples are ground in sample extraction buffer (1:10 w/w ratio) and incubated overnight at 4°C. After washing, monoclonal antibodies (MAb AS-1153/1) are added, followed by incubation and another wash. A secondary anti-mouse antibody conjugated with alkaline phosphatase (RAM-AP) is then added, followed by incubation and washing. Finally, a substrate solution of p-nitrophenyl phosphate is added, incubated and the results are measured by optical density at 405 nm. Positive results are indicated by absorbance values three times higher than the means of negative controls.

Reverse transcription-quantitative polymerase chain reaction (RT-qPCR) was performed using a duplex TaqMan assay employing primers and probes designed to simultaneously detect CBSV and UCBSV (Table S3). Portions (lobes) from the cassava leaf samples that were selected for the ELLA and ELISA tests were attached to blank newsprint papers arranged in layers of two to three and left to dry at ambient temperature. Approximately 30 mg of the dried leaf was subjected to RNA extraction using a modified cetyltrimethylammonium bromide method (Maruthi et al. 2002). RNA isolation was performed

using a cetyltrimethylammonium bromide (CTAB) protocol for cassava leaf samples (Maruthi et al., 2002), with minor modifications. Briefly, 100 mg of freshly collected leaf tissue was ground in 1 mL of CTAB buffer using a mortar and pestle. Seven hundred and fifty microliters of the resulting homogenate were mixed with an equal volume of chloroform: isoamyl alcohol (24:1). After phase separation, RNA was precipitated with 0.6 volumes of isopropanol from the recovered 500 µL aqueous phase. The RNA pellet was resuspended in 0.1× Tris-EDTA (TE) buffer (Invitrogen), and its quantity and quality were assessed using a NanoDrop spectrophotometer (ND2000; Thermo Fisher Scientific). RNA was stored at −20 °C for short-term use or at −80 °C for long-term storage. The real-time duplex RT-qPCR reaction mixture contained the components listed in Table S4.The duplex assay was performed using Agilent's AriaMx qPCR system (Agilent Technologies, Santa Clara, CA, USA) with the following thermocycling program: 48 °C for 30 minutes (cDNA synthesis), 95 °C for 10 minutes (initial denaturation and reverse transcriptase inactivation), followed by 40 cycles of 95 °C for 15 seconds, 52 °C for 20 seconds, 55 °C for 15 seconds, and 60 °C for 40 seconds (fluorescence data collection at this step). The quantification cycle values (Cq) were generated using the onboard AriaMx Software version 2.1.

Table S1 – Comparison of ELLA current with standard laboratory tests for CBSD detection.

Sample No.	Variety	CBSD stage	ELLA peak current (µA)*	qPCR quantification cycle (Cq)**	ELISA absorbance***
1	Albert	1	2.65	0.0	0.451
2	Albert	3	1.52	0.0	1.378
3	Albert	3	0.99	0.0	0.667
4	Albert	1	1.18	0.0	0.441
5	Albert	3	1.19	0.0	0.384
6	Albert	3	4.52	0.0	0.435
7	Albert	CMD	0.76	0.0	0.867
8	Albert	1	0.86	0.0	0.637
9	Albert	3	4.32	32.3	1.144

10	Albert	3	5.15	13.1	1.162
11	Albert	1	2.77	0.0	0.395
12	Albert	3	0.93	0.0	0.436
13	Albert	3	4.2	14.2	1.608
14	Albert	CMD	1.73	0.0	0.687
15	Albert	1	2.48	0.0	0.728
16	Albert	3	5.23	12.7	1.214
17†	Albert	3	3.41	13.5	0.563
18	Kiroba	1	2.52	0.0	0.535
19	Kiroba	3	12.58	31.8	1.798
20	Kiroba	3	5.28	15.3	0.857
21	Kiroba	1	1.1	0.0	0.714
22	Kiroba	3	5.24	0.0	1.256
23	Kiroba	3	1.81	0.0	0.57
24	Kiroba	1	0.54	0.0	0.381
25	Kiroba	2	2.73	0.0	0.375
26	Kiroba	1	0.74	0.0	0.412
27	Kiroba	2	3.25	0.0	0.642
28	Kiroba	1	1.9	0.0	0.45
29	Kiroba	3	8.44	0.0	1.066
30	Kiroba	3	4.69	0.0	0.728
31	Nyoku-Agbeli	1	3.25	0.0	0.686
32	Nyoku-Agbeli	3	11.85	13.3	0.943
33	Nyoku-Agbeli	3	6.84	0.0	0.938
34	Nyoku-Agbeli	1	1.04	0.0	0.619
35	Nyoku-Agbeli	3	10.68	15.1	0.845
36	Nyoku-Agbeli	3	4.52	0.0	0.937
37	Nyoku-Agbeli	1	0.57	0.0	0.539
38	Nyoku-Agbeli	CMD	1.02	0.0	0.698
39	Nyoku-Agbeli	2	12.23	11.9	1.575
40	Nyoku-Agbeli	2	2.42	0.0	0.353
41	Fufu-Bankye	1	4.28	13.1	1.015
42	Fufu-Bankye	2	5.65	20.1	1.543
43	Fufu-Bankye	1	1.14	0.0	0.472
44	Fufu-Bankye	3	14.22	4.3	0.936
45	Fufu-Bankye	3	13.16	10.8	1.081
46	UCC2001-053	1	3.5	0.0	0.461
47	UCC2001-053	3	4.76	12.7	1.638
48	UCC2001-053	3	10.94	0.0	0.526
49	UCC2001-053	1	3.16	0.0	0.464

50	UCC2001-053	3	7.24	18.2	1.506
51	UCC2001-053	3	8.55	11.4	0.843
52	Albert (Old)	1	1.54	22.6	0.454
53	Albert (Old)	4	7.63	15.9	1.088
54	Albert (Old)	4	5.18	13.0	1.815
55	Albert (Old)	4	29.41	15.6	1.864
56	Albert (Old)	1	8.03	21.7	1.653
57	Albert (Old)	4	1.74	16.7	0.711
58	Albert (Old)	4	2.21	13.2	0.474
59	Albert (Old)	4	4.22	13.3	1.777
60	Kiroba (Old)	1	3.62	18.6	0.911
61	Kiroba (Old)	3	9.29	14.7	1.039
62	Kiroba (Old)	3	13.46	14.5	1.435
63	Kiroba (Old)	1	6.49	23.1	1.322
64	Kiroba (Old)	3	8.79	17.8	1.761
65	Kiroba (Old)	3	11.83	19.3	0.962
66	Kiroba (Old)	1	7.01	20.2	1.564
67	Kiroba (Old)	3	8.68	19.0	1.04
68	Kiroba (Old)	3	15.11	19.0	0.9
69	Kiroba (Old)	1	8.64	21.5	0.92
70	Kiroba (Old)	3	13.65	19.8	1.737
71	Kiroba (Old)	3	13.47	19.7	1.504
72	Albert (Old)	1	1.39	0.0	0.433
73	Albert (Old)	3	2.4	0.0	0.491
74	Albert (Old)	3	11.25	0.0	0.946
75	Albert (Old)	1	3.35	0.0	0.6
76	Albert (Old)	3	0.73	0.0	0.743
77	Albert (Old)	3	1.73	0.0	0.534
78	Albert (Old)	1	0.83	0.0	0.413
79	Albert (Old)	4	7.87	0.0	0.955
80	Albert (Old)	4	3.33	0.0	0.698
81	Albert (Old)	4	0.78	0.0	0.633
82	Albert	1	2.76	0.0	0.462
83	Albert	2	2.34	0.0	0.417
84	Albert	2	2.12	0.0	0.458
85	Albert	2	8.07	17.9	1.18
86	031	1	2.11	0.0	0.657
87	031	5	1.98	0.0	0.401
88	031	5	10.67	0.0	1.472
89	031	5	14.15	17.0	1.591

90	031	5	22.7	15.6	0.979
91†	031	1	3.84	25.1	0.568
92	031	5	9.35	22.3	0.881
93	031	5	17.01	17.2	0.91
94	031	5	31.79	16.5	1.666
95	031	5	30.46	17.2	1.187
96	031	1	1.42	0.0	0.503
97	031	3	2.27	0.0	0.674
98	031	3	21.53	15.6	1.027
99	UKG 09-152	1	3.2	0.0	0.338
100	UKG 09-152	4	15.5	23.3	1.166
101	UKG 09-152	4	24.44	19.9	1.617
102	UKG 09-152	4	17.01	15.8	1.98
103	UKG 09-152	1	13.8	24.8	1.439
104	UKG 09-152	4	16.7	18.0	1.622
105	UKG 09-152	4	12.37	15.9	1.234
106	UKG 09-152	4	15.04	17.1	1.475
107	UKG 09-152	1	1.86	0.0	0.783
108	UKG 09-152	3	10.05	17.5	0.911
109	UKG 09-152	3	11.91	17.1	1.122
110	Albert	1	2.61	0.0	0.567
111	Albert	2	2.91	22.8	0.525
112	Albert	1	1.09	0.0	0.694
113†	Albert	2	5.53	21.0	0.602
114	Albert	1	9.48	0.0	0.752
115	Albert	2	3.86	22.2	0.644
116	Albert	2	4.65	17.9	1.122
117	Kiroba	3	9.76	17.0	1.823
118	Albert	3	17.81	16.8	1.672
119	TME 14	3	10.66	16.8	1.342
120	TME 204	3	7.54	16.2	1.156
121	Albert	1	1.19	0.0	0.547
122	Kiroba	1	4.03	0.0	0.634
123	TME 204	3	5.14	17.1	0.823
124	Albert	3	6.29	17.3	1.042
125	Albert	1	1.05	0.0	0.531
PC100%	-	-	11.04	10.1	2.341
PC50%	-	-	10.27	-	1.230
PC25%	-	-	9.01	-	0.764
PC10%†	-	-	6.55	-	0.341

PC5%†	-	-	4.58	-	0.324
NC	-	-	2.51	0.0	0.250

*Current values > 3.4 µA are considered positive for CBSD; **Cq values > 0 are considered positive for CBSD; ***Absorbance values > 0.75 are considered positive for CBSD (3x NC signal); PC = positive control; NC = Negative control; CMD = Plant infected with Cassava Mosaic Disease; † = samples positives for qPCR and ELLA but negative for ELISA, highlighting that ELLA could detect low title samples.

Table S2 – Cost of materials for one fully assembled ELLA platform.

Part	Cost* (USD)
Antibodies and Buffers	0.40
Nanoparticles	0.10
Nitrocellulose	0.08
Pads and backing card	0.04
3D printed cassette	0.03
Electrodes	0.03
SIC4341 Silicon Craft Chip	0.20
Capacitors	0.01
PCB	0.01
**Total**	**US$ 0.90**

*Prices are converted from GBP using the prevailing exchange rate, subject to change. SIC4341 chips were obtained from Silicon Craft PLC and the rest of the electronic components were purchased from Mouser Electronics.

Table S3 - Primer and Probe Information

Name of oligo	Sequence (5'-3' orientation)	Target Region	5'- Modification	5'- Modification
UCBSV CP_F18	CAGTYGARYTRAATTCTCA	UCBSV CP		
UCBSV CP_R18	GAGKATDGCTTGRCTYGA	UCBSV CP		
UCBSV CP_prb18	TCATWAYYT/ZEN/TCCTCAT WGTYGGYAAT	UCBSV CP	/56-FAM/	/ZEN//3IaBkF Q/
CBSV CP_F18	AGTGGTTRGGTATTGACTTC	CBSV CP		

CBSV		CBSV		
CP_R18	GCAGCTAAAGCAAACAAC	CP		
CBSV	TTCTACCAACATTAGCAGCCA	CBSV		
CP_prb18	RTATT	CP	/5HEX/	/3BHQ_1/

Table S4 - Reaction mixture for the duplex real-time RT-qPCR assay

Component	×1 Reaction (uL)	Initial concentration	Final concentration	Units
PCR Water	7.646	-		
10× PCR Buffer II	2.0	10	1	×
MgCl2	3.2	25	4	mM
dNTPs	1.0	10	0.5	mM
CBSV CP-F18/R18 mix	0.8	7.5	0.3	µM
CBSV CP_prb18	0.4	5	0.1	µM
UCBSV CP_F18/R18 mix	0.936	7.5	0.351	µM
UCBSV CP_prb18	0.8	5	0.2	µM
0.1× ROX	2.0	0.1	0.01	x
5.0 U/uL Taq DNA Poly.	0.15	5	0.8	Units
200 U/uL M-MLV RT	0.068	200	14	Units
Template	1	-		-
Total	20			

References

1. Texas Instruments. Coil Designer. https://webench.ti.com/wb5/LDC/#/spirals (2024).
2. Wang, Y., Limon-Petersen, J. G. & Compton, R. G. Measurement of the diffusion coefficients of [Ru(NH3)6]3+ and [Ru(NH3)6]2+ in aqueous solution using microelectrode double potential step chronoamperometry. *J. Electroanal. Chem.* **652**, 13–17 (2011).
3. Maruthi, M.N.; Colvin, J.; Seal, S.; Gibson, G.; Cooper, J. Co-adaptation between cassava mosaic geminiviruses and their local vector populations. *Virus Res. 2002*, **86**, 71–85.
